# Nicotinamide phosphoribosyltransferase in NAD^+^ metabolism: physiological and pathophysiological implications

**DOI:** 10.1038/s41420-025-02672-w

**Published:** 2025-08-08

**Authors:** Weijia Zhang, Haoyu Ren, Wangwang Chen, Bo Hu, Chao Feng, Peishan Li, Yufang Shi, Jiankai Fang

**Affiliations:** 1 The Fourth Affiliated Hospital of Soochow University, Institutes for Translational Medicine, State Key Laboratory of Radiation Medicine and Protection, Suzhou Medical College of Soochow University, Suzhou, Jiangsu 215123 China; 2 Laboratory Animal Center, Suzhou Medical College of Soochow University, Suzhou, Jiangsu 215123 China; 3 Department of Experimental Medicine and Biochemical Sciences, TOR, University of Rome “Tor Vergata”, 00133 Rome, Italy; 4 Shanghai Institute of Nutrition and Health, Chinese Academy of Sciences, Shanghai, 200025 China

**Keywords:** Metabolic disorders, Cancer metabolism

## Abstract

Nicotinamide adenine dinucleotide (NAD⁺) is a critical coenzyme involved in cellular metabolism, energy balance, and various physiological processes. Nicotinamide phosphoribosyltransferase (NAMPT) is a key rate-limiting enzyme in NAD⁺ synthesis, regulating the NAD⁺ regeneration pathway. This review summarizes the multiple roles of NAMPT in both physiological and pathological states, particularly in cellular stress, aging, metabolic disorders, and cancer. We first describe the central role of NAMPT in NAD⁺ synthesis and explore how NAD⁺ levels are regulated through NAMPT to control cellular functions and metabolic adaptation. Second, we analyze the pathological roles of NAMPT in aging and related diseases, highlighting how NAD⁺ depletion leads to mitochondrial dysfunction, DNA damage, and immune system dysregulation. Notably, NAMPT exacerbates cancer immune evasion mechanisms by influencing immune cell functions and the metabolic environment of tumors. We also discuss the potential of NAMPT as a therapeutic target, particularly through NAD⁺ precursor supplementation or the use of NAMPT activators and inhibitors to modulate NAD⁺ metabolism in aging, metabolic diseases, and cancer. Future research should focus on exploring the functional differences of NAMPT in various tissues and its therapeutic potential in disease treatment.

## Facts

- NAMPT regulates NAD^+^ biosynthesis through the salvage pathway, particularly during stress, aging, and inflammation.
- NAMPT and NAD^+^ decline with aging, leading to mitochondrial dysfunction, DNA damage, and metabolic disorders.
- NAMPT modulates tumor metabolism and immune evasion through extracellular NAMPT, influencing tumor progression.

## Open Questions

- What are the specific functions and mechanisms of NAMPT in different tissues, and how does it precisely regulate NAD^+^ metabolism?
- How does NAMPT balance its dual role in aging, both as a regulator of cellular metabolism and a potential contributor to aging-related diseases?
- What is the exact role of NAMPT in cancer immune evasion, and how can it be effectively combined with other treatments, such as immune checkpoint inhibitors?

## Introduction

Nicotinamide adenine dinucleotide (NAD⁺) is a fundamental coenzyme in cellular metabolism, essential for maintaining cellular energy homeostasis. It plays a pivotal role in redox reactions by accepting and donating electrons in key metabolic processes, including glycolysis, the tricarboxylic acid (TCA) cycle, oxidative phosphorylation (OXPHOS). In addition to its redox function, NAD⁺ is involved in non-redox signaling processes that regulate various cellular functions, including DNA repair, protein modifications, and the modulation of immune responses. The metabolism of NAD⁺ is highly dynamic, influenced by factors such as age, diet, and stress, and is tightly regulated by a network of biosynthetic, salvage, and degradative pathways [1, 2]. One key enzyme in NAD⁺ metabolism is nicotinamide phosphoribosyltransferase (NAMPT), which catalyzes the rate-limiting step in the NAD⁺ salvage pathway. This pathway is crucial for replenishing NAD⁺ pools, especially under conditions of high NAD⁺ consumption, such as during cellular stress and inflammation. Dysregulation of NAD⁺ metabolism is increasingly recognized as a contributing factor to the pathogenesis of numerous diseases, including metabolic disorders, neurodegenerative conditions, cancer, and age-related diseases. The decline in NAD⁺ levels with age and in certain pathological states has spurred interest in therapeutic strategies aimed at restoring NAD⁺ homeostasis. These include the supplementation of NAD⁺ precursors, such as nicotinamide riboside (NR) and nicotinamide mononucleotide (NMN), which have shown promise in preclinical models [3, 4]. This review explores the physiological roles of NAMPT and NAD⁺ metabolism in health. Furthermore, it addresses the pathophysiological consequences of NAD⁺ imbalance in diseases and discusses the therapeutic potential of NAD⁺ modulation.

## Physiological roles of NAMPT

### Cellular NAD^+^ metabolism

Since NAD^+^’s discovery in the early 20th century, significant progress has been made in elucidating its structure and biological functions. In 1906, British biochemists Arthur Harden and William John Young identified a small molecule that enhanced the rate of sugar fermentation in yeast, referring to it as a “coenzyme,” which was later recognized as NAD^+^ [5]. In 1936, German physiologist Otto Warburg demonstrated that NAD⁺ functions as a coenzyme for dehydrogenases in redox reactions within the cellular respiratory chain [6]. This discovery connected NAD⁺ to critical metabolic processes and stimulated extensive research into its role in human health. Subsequent studies determined the complete molecular structure of NAD⁺, confirming that it consists of adenine mononucleotide (AMP) and NMN, linked by a phosphodiester bond [7]. This structural elucidation provided a foundation for further exploration of NAD⁺ metabolism and its derivatives, including nicotinamide adenine dinucleotide phosphate (NADP⁺).

In biological systems, NAD primarily exists in its oxidized (NAD⁺) and reduced (NADH) forms, facilitating crucial redox reactions such as glycolysis, the TCA cycle, OXPHOS, fatty acid oxidation (FAO), and amino acid metabolism. During these processes, dehydrogenases transfer hydrogen atoms from substrate molecules to NAD⁺, reducing it to NADH. NADH then donates electrons to the mitochondrial respiratory chain, ultimately generating adenosine triphosphate (ATP) via OXPHOS, a vital process that powers diverse cellular functions [8, 9]. Beyond energy metabolism, NAD⁺ also serves as a substrate for several key enzymes. For instance, poly(ADP-ribose) polymerases (PARPs) utilize NAD⁺ during DNA damage repair, while sirtuins (silent information regulator proteins) use NAD⁺ for protein deacetylation, thereby influencing gene expression, aging, metabolism, and circadian rhythms. Additionally, NAD⁺ is degraded by CD38 and related enzymes, producing ADP-ribose, a molecule involved in calcium signaling and the immune regulation of inflammation [10–14].

**Fig. 1:**
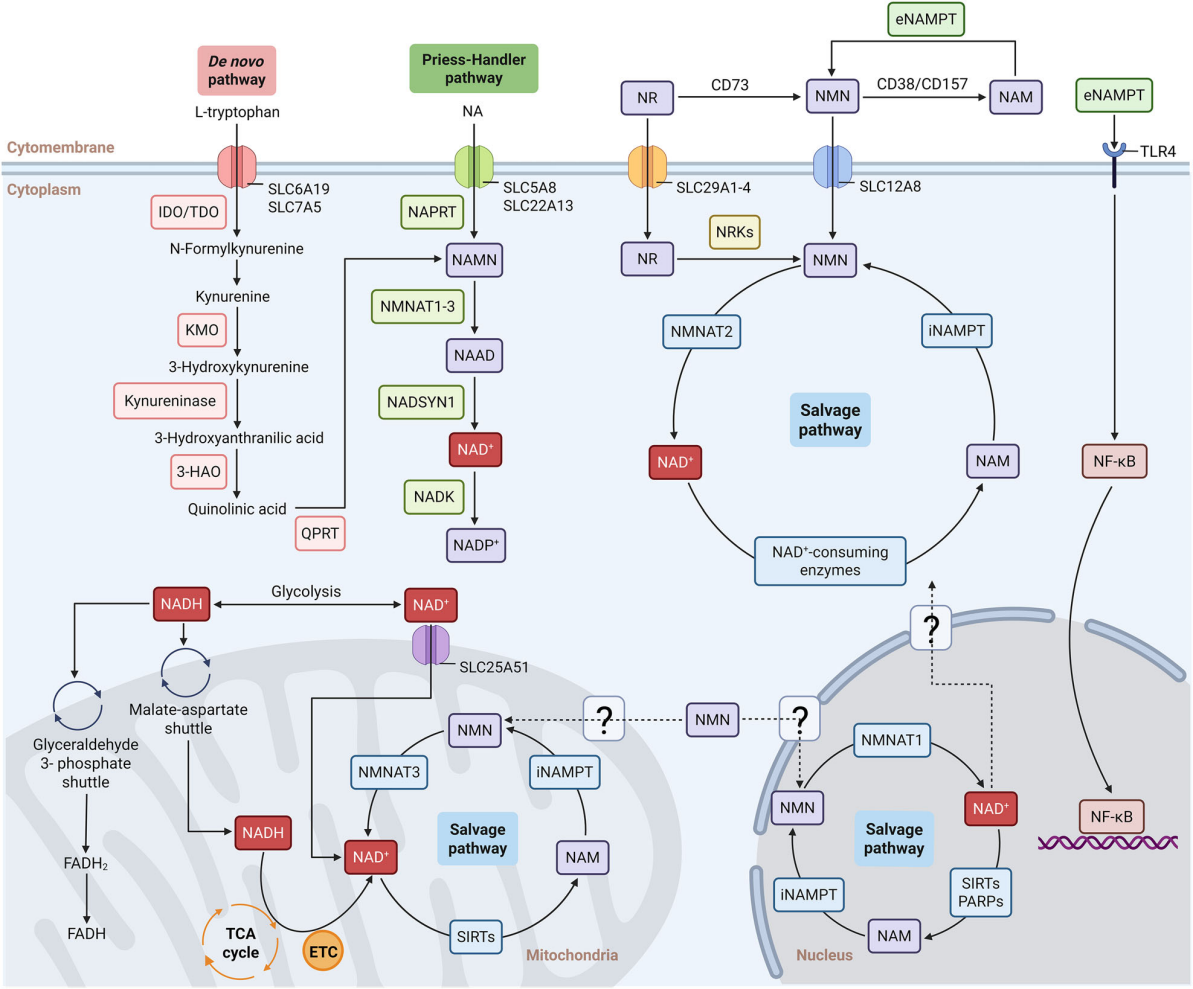
The synthesis and utilization of NAD^+^ within various cellular compartments, including the cytoplasm, mitochondria, and nucleus. In the cytoplasm, NAD⁺ is synthesized from precursors such as nicotinamide (NAM), nicotinic acid (NA), and tryptophan (Trp) through distinct metabolic pathways, including the Preiss-Handler pathway, the de novo synthesis pathway, and the salvage pathway—the latter being particularly predominant in mammalian cells. Within the mitochondria, NAD⁺ functions as a coenzyme for various dehydrogenases involved in the tricarboxylic acid (TCA) cycle and oxidative phosphorylation, playing a critical role in mitochondrial function and ATP production. In the nucleus, NAD⁺ serves as a substrate for PARP-mediated ADP-ribosylation and SIRT-mediated deacetylation, both of which are essential for the regulation of chromatin structure and the DNA damage response.

The biosynthesis of NAD⁺ is crucial for maintaining intracellular NAD⁺ homeostasis and occurs through three primary pathways: the salvage pathway, the de novo synthesis pathway, and the Preiss-Handler pathway [15–17]. Among these, the salvage pathway is the predominant mechanism in mammals for sustaining NAD⁺ levels. It primarily utilizes nicotinamide (NAM), NR, and NMN as precursors, with NAMPT serving as the rate-limiting enzyme in NAD⁺ synthesis. This pathway prevents excessive NAD⁺ depletion through a series of enzymatic reactions. In contrast, the de novo synthesis pathway converts tryptophan (Trp) into nicotinic acid mononucleotide (NAMN) via the kynurenine metabolic pathway, ultimately leading to NAD⁺ production. This pathway is regulated by various metabolic and immune signals and is closely linked to conditions such as cancer and inflammation-mediated psychiatric disorders [18, 19]. On the other hand, the Preiss-Handler pathway uses nicotinic acid (NA) as a precursor and relies on nicotinic acid phosphoribosyltransferase (NAPRT) for NAD⁺ synthesis. Unlike the other two pathways, the Preiss-Handler pathway mainly depends on dietary intake and is the primary route through which vitamin B3 supplementation boosts NAD⁺ levels [20]. Together, these biosynthetic pathways function in a complementary manner to maintain intracellular NAD⁺ homeostasis under both physiological and pathological conditions (Fig. 1).

### NAMPT-mediated NAD^+^ biosynthesis

NAD⁺ recycling through the NAM salvage pathway plays a crucial role in restoring NAD⁺ levels after its irreversible degradation, facilitated by various NAD⁺-consuming enzymes such as glycohydrolases (e.g., CD38, CD157, and SARM1), sirtuins, and PARPs [21]. While the specific functions and roles of NAD⁺ differ among these enzymes, all NAD⁺-consuming enzymes generate NAM as a by-product of NAD⁺ degradation. This NAM is then converted to NMN by the enzyme NAMPT, which is widely expressed and particularly upregulated in processes requiring high NAD⁺ levels, such as mesenchymal stem cell (MSC) activation and genotoxic stress [22, 23]. Furthermore, NAMPT expression is modulated by the circadian clock machinery (CLOCK–BMAL1), creating a feedback loop with SIRT1, which is linked to the circadian fluctuations of NAD⁺ levels in vivo [24, 25]. In mammals, NAMPT exists in two forms: intracellular NAMPT (iNAMPT) and extracellular NAMPT (eNAMPT). As an NAD⁺ biosynthetic enzyme, NAMPT functions by forming homodimers [26]. NAD⁺ is continuously synthesized, broken down, and recycled within the cell to maintain a stable intracellular concentration. However, under pathological conditions, this balance between catabolic and anabolic processes can be disrupted, causing NAD⁺ degradation to exceed the cell’s ability to synthesize NAD⁺ de novo or efficiently recycle and salvage NAM, thereby exacerbating disease phenotypes.

In addition to its role in regulating NAM salvage, eNAMPT was initially identified in pre-B cells and was originally termed “pre-B cell colony-enhancing factor” (PBEF) due to its ability to synergize with interleukin-7 (IL-7) and stem cell factor (SCF) in promoting pre-B cell colony formation [27]. eNAMPT is primarily derived from multiple biological sources, including circulatory system [28, 29], immune system [30–32], cerebrospinal fluid [33], adipose tissue [34, 35], liver [36], and cardiomyocytes [37], with adipose tissue playing a pivotal role in regulating systemic eNAMPT levels [35]. Notably, the secretion of eNAMPT does not follow the conventional endoplasmic reticulum-Golgi pathway; instead, it is predominantly released into the extracellular environment via exosomes and microvesicles [38]. Through circulation, eNAMPT exerts systemic effects by modulating NAD⁺ levels in distal organs, a process influenced by various physiological and pathological conditions, such as metabolic stress, inflammation, hypoxia, and aging [39]. Yoon et al. demonstrated that SIRT1-mediated deacetylation of lysine 53 (K53) enhances both the enzymatic activity and extracellular release of eNAMPT [40]. However, the precise regulatory factors and mechanisms governing its secretion remain to be fully elucidated. In the extracellular space, eNAMPT functions not only as a key regulator of NAD⁺ biosynthesis but also as an endogenous damage-associated molecular pattern (DAMP). It interacts with specific receptors, such as Toll-like receptor 4 (TLR4), or engages in signaling crosstalk, triggering pro-inflammatory cascade responses [41, 42]. The application of eNAMPT-neutralizing antibodies has been shown to effectively block its receptor interactions, leading to a reduction in pro-inflammatory cytokine production, attenuation of tissue damage, and alleviation of acute and chronic inflammation-related diseases, such as colitis and radiation pneumonitis [43, 44]. Furthermore, eNAMPT levels are significantly elevated in the plasma of patients with various autoimmune diseases (e.g., systemic lupus erythematosus) [45], metabolic disorders (e.g., obesity, diabetes) [46], aging [47], and malignancies [48]. Consequently, eNAMPT is considered a potential biomarker for early disease diagnosis and monitoring disease progression. Despite these advances, the precise roles of eNAMPT in different pathological conditions remain incompletely understood. Further investigations into its regulatory mechanisms, secretion dynamics, and signaling pathways may provide valuable insights into its clinical applications and pave the way for novel therapeutic strategies targeting eNAMPT-related diseases.

## Pathophysiological roles of NAMPT

### NAMPT and obesity

Fat serves as an essential energy reserve, playing a crucial role in the survival of many species during periods of food scarcity or environmental changes. Paradoxically, in contemporary society, characterized by sedentary lifestyles and unhealthy diets leading to caloric surplus, this detrimental trait has become associated with a range of health issues arising from metabolic disturbances, including lipid accumulation, insulin resistance, inflammation, and organelle stress [49]. Numerous preclinical and clinical studies have identified obesity as a significant risk factor for metabolic diseases and organ complications, often linked to chronic inflammation in adipose tissues [50]. Obesity induces specific physiological responses and alters adipokine secretion patterns in adipose tissues, which recruit and activate immune cells, such as macrophages, while promoting adipocyte hypertrophy [51]. This, in turn, results in the release of pro-inflammatory cytokines, including TNF-α and IL-6. Excess lipids also accumulate in non-adipose tissues, such as the liver, muscle, and pancreas, leading to lipotoxicity. This disrupts cellular organelles, causing the release of reactive oxygen species (ROS) and pro-inflammatory mediators, which intensify systemic inflammation [52]. Chronic low-grade inflammation contributes to the development of insulin resistance by impairing insulin signaling pathways, ultimately disrupting glucose homeostasis [53]. A growing body of evidence suggests that, in response to glucose or factors inducing insulin resistance (e.g., IL-6, dexamethasone, growth hormone, tumors), adipose tissue may function as an endocrine organ, producing and secreting various adipokines [54, 55]. NAMPT, which exhibits insulin-mimetic effects, is expressed in visceral adipose tissue in both humans and mice, with its plasma levels increasing as obesity develops [56].

The role of NAMPT in the development of obesity is multifaceted, playing a critical role in regulating adipose tissue plasticity, food intake, and systemic glucose balance. In a high-fat diet (HFD)-induced obesity mouse model with adipose-specific *Nampt* knockout (FANKO), FANKO mice were completely resistant to obesity, exhibited significantly reduced food intake, and demonstrated improved glucose tolerance compared to control littermates. However, HFD-fed FANKO mice showed a tendency for adipose tissue fibrosis, were unable to expand healthily, and exhibited significantly reduced mitochondrial respiratory capacity [57], implying that NAMPT-mediated NAD^+^ biosynthesis is crucial for cellular fitness in adipose tissue. NAD⁺-dependent deacetylases, particularly sirtuins, are key regulators of obesity-associated adipose tissue remodeling [58]. Adipose tissue can respond to chronic energy excess by specifically inhibiting the metabolic components of NAD⁺/SIRTs. A reduction in NAD⁺ levels decreases SIRT1 activity, diminishing adipocyte sensitivity to insulin, thereby exacerbating adipose accumulation and metabolic disturbances [59]. Moreover, the interaction between astrocytes and neurons in the hypothalamus plays an important role in regulating body weight and peripheral nutrient metabolism [60, 61]. NAMPT expression in the hypothalamus is downregulated at both the mRNA and protein levels following HFD induction [62], while lipid overload disrupts hypothalamic circuits, triggers inflammation, and hinders energy homeostasis regulation, ultimately leading to weight gain [63, 64]. A recent study demonstrated that saturated fatty acids modulate the effects of high-fat diets on body weight through activation of CD38 in hypothalamic astrocytes via the NAMPT-dependent NAD⁺ supplement. This activation upregulates pro-inflammatory responses, disrupts Ca²⁺ signaling, and alters responses to metabolic hormones such as insulin and leptin, ultimately impairing astrocyte function. Inhibition of this pathway mitigates hypothalamic inflammation and slows the progression of obesity [65].

The suprachiasmatic nucleus (SCN) of the hypothalamus is the body’s primary biological clock, responsible for sensing circadian changes and regulating circadian rhythms. One of the key metabolic signals is eNAMPT, which regulates NAD⁺ biosynthesis—a system that can be significantly disrupted by obesity. Studies have shown that obese mice exhibit notably diminished circadian oscillations in the blood eNAMPT-hypothalamic NAD⁺-FOXO1 axis following exercise and energy expenditure [29]. Both brown adipose tissue (BAT), responsible for caloric expenditure, and white adipose tissue (WAT), responsible for fat storage, require NAD⁺ to maintain the amplitude of the core circadian clock [66]. In diet-induced obesity models in rodents, NAD⁺ levels in vivo decrease in a non-rhythmic manner, and timed NAD⁺ supplementation can influence the oscillatory phase of the hepatic molecular clock. Increasing NAD⁺ levels prior to the active phase improves metabolic markers, including body weight, glucose, and insulin tolerance, and hepatic inflammation. However, elevating NAD⁺ levels before the resting phase may selectively inhibit these responses via a specific mechanism regulated by CLOCK/BMAL1-dependent AMPK/SIRT1 expression [67]. Furthermore, synchronization with NAD⁺-SIRT1 circadian rhythms through enhanced aerobic training promotes MFN2-mediated mitochondrial fusion by activating the BMAL1-PER2-SIRT1-PPARα axis in the skeletal muscle of diabetic mice. This approach is more effective in improving glycemic control and insulin resistance [68].

In conclusion, both systemic and adipose tissue-specific NAMPT play crucial roles in regulating the development of obesity. NAMPT influences fat accumulation by modulating adipose tissue metabolism and systemic glucose homeostasis, and is also closely linked to hypothalamic energy balance and circadian rhythms. Obesity exacerbates metabolic disturbances by disrupting these mechanisms.

### NAMPT and non-alcoholic fatty liver

Non-alcoholic fatty liver disease (NAFLD) has emerged as one of the most prevalent chronic liver diseases worldwide, with its incidence rising at an alarming rate [69]. This trend is strongly linked to the widespread adoption of high-calorie diets, poor lifestyles, and the increasing prevalence of obesity and metabolic disorders. NAFLD typically begins with simple lipid accumulation in the liver, and as the disease progresses, it may advance to non-alcoholic steatohepatitis (NASH), which can further deteriorate into liver fibrosis, cirrhosis, and ultimately hepatocellular carcinoma, with inflammation being an integral part of the disease progression [70, 71]. Elevated levels of eNAMPT and inflammatory cytokines have been observed in both liver tissue and plasma of human NAFLD patients, as well as in NAFLD model mice, suggesting a close association between eNAMPT levels and liver inflammation [41]. However, oral supplementation with NAD⁺ precursors, such as NR and NMN, has been shown to reduce hepatic lipid accumulation and mitochondrial oxidative stress by enhancing hepatic NAD⁺ levels in preclinical NAFLD models. Furthermore, NAD⁺ supplementation has been demonstrated to prevent the progression of NAFLD to NASH, a process associated with the downregulation of inflammation-driven hepatic stellate cell activation and amelioration of hepatic fibrosis [72]. The differential effects of eNAMPT and NAD⁺ precursors in regulating liver inflammation highlight the complexity of NAD^+^ metabolism in immune modulation.

NAD⁺-dependent deacetylase SIRT2 acts as a key negative regulator in NAFLD and related metabolic disorders. A recent study found that the circadian expression of both SIRT2 and BMAL1 was suppressed in palmitic acid-treated non-cancerous hepatocytes. Mechanistically, palmitic acid represses SIRT2 transcription by disrupting the chromatin binding of BMAL1 to its promoter region. However, ectopic expression of BMAL1 and the activation of SIRT2 through NR supplementation effectively attenuate palmitic acid-induced liver inflammation and lipotoxicity in hepatocytes [73]. SIRT2 stabilizes the expression of hepatocyte nuclear factor 4α (HNF4α) by binding to and deacetylating its lysine 458 residue, a function essential for SIRT2 activity both in vitro and in vivo [74]. Furthermore, activation of HNF4α using N-trans-caffeoyltyramine (NCT) increases mitochondrial mass and fatty acid oxidation, leading to increased NAD⁺ levels, weight loss, and reduced hepatic steatosis [75]. A knockdown screen using shRNA revealed that the NAD⁺-dependent deacetylase SIRT2 interacts with Fndc5 to induce its deubiquitination and deacetylation, a process potentially dependent on the lysine residues K127/131 and K185/187/189 of Fndc5. When NAD⁺ enhancement with NR in NAFLD patients and animal models, elevated plasma levels of Fndc5/irisin were observed in both mice and humans, along with increased expression of Fndc5 in skeletal muscle, adipose, and hepatic tissues in mice. When *Fndc5*^*-/-*^ mice were used to model HFD-induced NAFLD, the positive effects of NR supplementation were diminished. However, slow infusion of recombinant Fndc5/irisin led to a significant reduction in the pathological phenotype of NAFLD in the mouse model. This suggests that NR reduces lipid stress-induced ubiquitylation of Fndc5, thereby enhancing the stability of the Fndc5 protein [76].

A recent study demonstrated that the hepatic mitochondrial NAD⁺ transporter SLC25A47 activates AMPKα, mediating lipid metabolism and tumorigenesis. Slc25a47 deficiency was accompanied by decreased activity of the NAD⁺-dependent deacetylase SIRT3, which suppressed AMPKα phosphorylation and led to an increased accumulation of nuclear sterol regulatory element-binding proteins. This, in turn, elevated fatty acid and cholesterol biosynthetic activities, promoting hepatocellular carcinoma tumorigenesis and development through the activated mammalian target of rapamycin (mTOR) cascade [77]. Furthermore, induction of the NAMPT/NAD⁺/SIRT1 axis in a methionine-choline-deficient diet-induced mouse model of NASH resulted in a significant reduction in liver inflammation, accompanied by a decrease in total bile acid levels throughout the enterohepatic circulation and a shift in the bile acid synthesis pathway from the classical to an alternative pathway, leading to a reduction in pro-inflammatory 12-OH bile acid production [78]. In NAFLD and related diseases, miR-873-5p serves as a key regulator of NAD⁺ metabolism and SIRT1 deacetylase activity. Inhibition of miR-873-5p promotes the NAD⁺ salvage pathway and restores SIRT1 acetylation, while regulating downstream levels of NF-κB and FXR (two known SIRT1 substrates), thus maintaining hepatic bile acid homeostasis and attenuating the inflammatory response [79]. A study reported that *CD38*^*-/-*^ mice were also able to reduce HFD- or oleic acid-induced lipid accumulation and oxidative stress, inhibit NOX4 expression in liver tissues and hepatocytes, and simultaneously increase the expression of PPARα, CPT1, ACOX1, and SOD2. These effects were reversed by Ex527 (SIRT1 inhibitor) and 3-TYP (SIRT3 inhibitor), suggesting that the resistance to lipid accumulation in CD38-deficient mice may depend on NAD⁺/sirtuin-mediated enhancement of fatty acid β-oxidation and inhibition of oxidative stress in liver tissues [80]. Additionally, methylation-controlled J protein deficiency enhances mitochondrial activity, leading to increased NAD⁺ levels, which improves fatty acid oxidation and reduces hepatic lipid accumulation. This effect is linked to changes in the gut microbiota, particularly the increase in the Dorea genus, which promotes NAD⁺ biosynthesis and SIRT1 activity, contributing to the protective phenotype against NASH [81].

Taken together, NAD⁺ metabolism and sirtuins, particularly SIRT1 and SIRT2, play pivotal roles in the pathogenesis of NAFLD. Supplementation with NAD⁺ precursors can effectively reduce hepatic fat accumulation, alleviate mitochondrial oxidative stress, and enhance liver function. Additionally, sirtuins are critical regulators of metabolic processes, energy homeostasis, and anti-inflammatory responses. Alterations in their activity are strongly associated with the progression of NAFLD. Therefore, modulation of NAD⁺ metabolism and sirtuins presents promising therapeutic targets for NAFLD, and future research may provide new insights into clinical treatments.

### NAMPT and type 2 diabetes mellitus

Several studies have shown that obesity, particularly abdominal obesity, is an independent risk factor for type 2 diabetes [82]. Abdominal lipolysis occurs more rapidly than in other body regions, leading to the release of free fatty acids that directly disrupt insulin signaling, reduce glucose uptake and utilization in peripheral tissues (e.g., skeletal muscle and adipose tissue), and also promote abnormal triglyceride accumulation and excessive glucose isomerization in the liver, resulting in elevated blood glucose levels [83, 84]. Insulin resistance, coupled with a systemic low-grade inflammatory response, further disturbs metabolic homeostasis, ultimately contributing to the development of type 2 diabetes. Recent research has highlighted the dysfunction of adipose tissue as an endocrine organ that secretes various adipocytokines as a key molecular mechanism in the onset of diabetes [85]. Among these molecules, eNAMPT has emerged as a promising therapeutic target, being significantly elevated in patients with type 2 diabetes and corresponding mouse models compared to healthy controls. It directly influences pancreatic β-cell dysfunction and stimulates insulin secretion [86–89].

The onset of diabetes is often associated with chronic low-grade inflammation and oxidative stress, both of which underlie common diabetic complications (such as cardiovascular disease, myopathy, and neuropathy) [90]. It is worth mentioning that NAMPT plays a complex role in the progression of these complications. Under normal physiological conditions, the dimeric form of NAMPT helps maintain NAD⁺ levels, which in turn supports pancreatic β-cell function. However, as blood glucose levels rise, eNAMPT undergoes depolymerization into a less active monomeric form, which mediates β-cell dysfunction through a NAD⁺-independent and pro-inflammatory mechanism, thereby inducing a diabetic phenotype [91]. Due to mitochondrial dysfunction, diabetic patients often exhibit muscle weakness, reduced endurance, increased fatigability, and glycolysis dependence [92]. In a high-glucose-induced zebrafish model of type 2 diabetes, chronic high glucose exposure led to a progressive decline in mitochondrial function, mediated through the activation of the miR-139-5p/NAMPT pathway. This activation exacerbated diabetic myopathy [93]. Glyoxalase 1 (GLO1), a key detoxification enzyme involved in the formation of advanced glycation end-products (AGEs), is responsible for the removal of methylglyoxal (MG), a toxic intermediate. In the absence of GLO1, MG accumulates in the body, potentially contributing to insulin resistance and the development of diabetes. Edwin’s team found that GLO1 expression in the skeletal muscle of type 2 diabetic patients is regulated by NAMPT/SIRT, and supplementation with NR prevented the reduction of GLO1 expression and activity [94, 95]. Additionally, another study demonstrated that activation of NAMPT using the NAMPT agonist P7C3 significantly mitigated the progression of type 2 diabetes in *db/db* mice, while also improving skeletal muscle function [96]. The incidence of cardiovascular disease is notably higher in individuals with type 2 diabetes, and NAMPT indirectly influences cardiovascular health through its regulation of NAD⁺ levels, particularly by modulating endothelial function, vascular smooth muscle cell proliferation, and antioxidant activity [42, 97]. In a mouse model of type 2 diabetes induced by low-dose streptozotocin combined with a high-fat diet, diabetic *Nampt*^*+/-*^ heterozygous mice exhibited significantly improved coronary flow velocity reserve (CFVR), increased left ventricular capillary density, and enhanced coronary endothelium-dependent diastolic response (EDR). Inhibition of NAMPT using a neutralizing monoclonal antibody to eNAMPT or FK866 also significantly increased CFVR in diabetic mice. Additionally, administration of the eNAMPT monoclonal antibody upregulated the expression of angiogenesis- and EDR-related genes in cardiac endothelial cells of diabetic mice [97]. Moreover, eNAMPT can activate NLRP3 inflammasomes via a NAMPT-binding TLR4-mediated signaling pathway, leading to vascular dysfunction and triggering the release of IL-1β, a key mediator of endothelial injury [42].

The accumulation of advanced glycation end products (AGEs) is a common pathological feature in diabetes. AGEs bind to cell surface receptors for advanced glycation end products, activating inflammatory and oxidative stress responses, which further damage neural tissues [98]. In this context, extracellular levels of eNAMPT, particularly the monomeric form, are significantly elevated and positively correlated with increased inflammatory cytokine expression in *db/db* mice with middle cerebral artery occlusion/reperfusion [99]. Another study revealed the role of NAMPT in Neuro2a cells under high-glucose (HGC) and oxygen-glucose deprivation (OGD) conditions, which mimic diabetic cerebral infarction. Treatment with the NAMPT agonist P7C3-A2 suppressed the elevation of FOXO3a and LC3-II levels and effectively inhibited the reduction in cell viability induced by HGC/OGD [100]. Additionally, oxidative stress plays a pivotal role in the progression of diabetic nephropathy. NAD⁺-dependent deacetylase SIRT3 can attenuate albuminuria, mitigate glomerular injury, and reduce podocyte damage by activating SOD2 and restoring the expression of PGC-1α in glomerular cells [101].

**Fig. 2:**
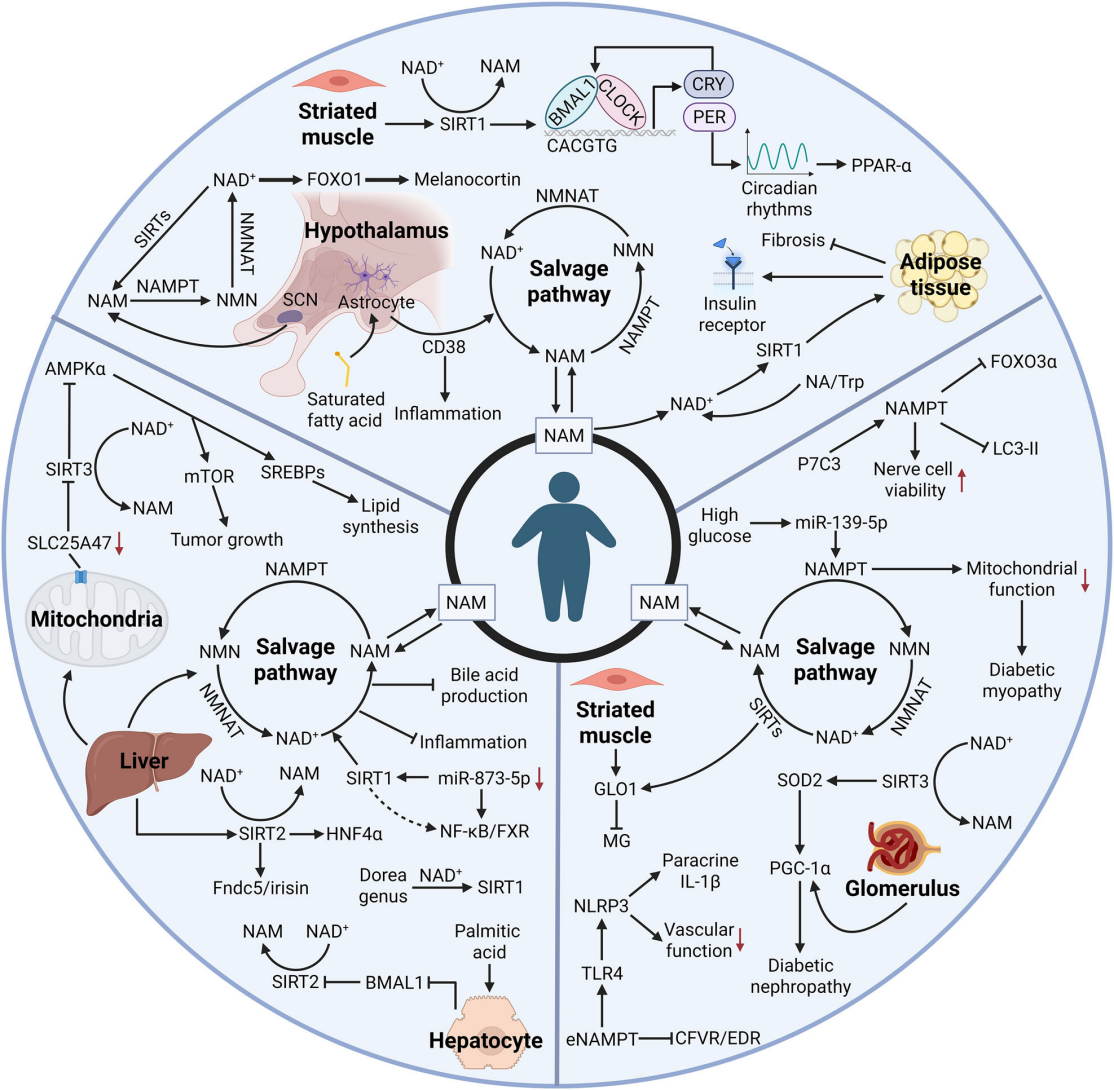
Central role of NAMPT in metabolic homeostasis and related diseases. NAMPT regulates NAD⁺ biosynthesis, influencing adipose tissue plasticity, food intake, systemic glucose homeostasis, and circadian rhythm synchronization. In obesity, dysregulated NAMPT expression contributes to chronic low-grade inflammation in adipose tissues, leading to insulin resistance, lipid accumulation, and multi-organ dysfunction. In non-alcoholic fatty liver disease (NAFLD), NAMPT-mediated disruption of NAD⁺ metabolism is closely associated with hepatic lipid deposition, inflammation, and fibrosis progression. NAD⁺ precursor supplementation improves liver function and attenuates NAFLD pathology through the activation of sirtuin-dependent pathways. In type 2 diabetes mellitus, elevated eNAMPT levels impair pancreatic β-cell function and promote inflammation and oxidative stress, exacerbating metabolic dysfunction and complications such as cardiovascular disease, neuropathy, and muscle impairment. The regulatory network of NAMPT in both central and peripheral tissues underscores its significance as a potential therapeutic target in the treatment of metabolic disorders.

The above findings suggest that eNAMPT not only directly regulates pancreatic β-cell function but also contributes to the pathogenesis of diabetes mellitus by enhancing insulin secretion and mitigating inflammatory responses. Moreover, in the context of chronic inflammation and oxidative stress, NAMPT is closely linked to diabetic complications, including muscle dysfunction, neuropathy, and cardiovascular disease. Selectively targeting monomeric eNAMPT may represent a promising therapeutic strategy for alleviating type 2 diabetes and its associated complications (Fig. 2).

### NAMPT and aging

Extensive animal studies and clinical investigations have demonstrated age-dependent declines in both NAMPT and NAD⁺ levels during aging and age-related pathologies. In adult-onset aging-associated disorders, tissue-specific NAD⁺ concentrations exhibit reductions ranging from 10% to 50% across various organs [102]. The progressive deterioration of NAMPT expression and enzymatic activity in vivo drives NAD⁺ depletion, compromising cellular energy metabolism and impairing DNA repair mechanisms. This NAD⁺-dependent functional decline ultimately contributes to the pathogenesis of aging-related diseases.

Age-related disorders are linked to NAMPT-mediated dysregulation of cellular energy metabolism. Enhanced NAMPT-driven NAD⁺ salvage pathway ameliorates aging skeletal muscle function through multiple mechanisms: mitigating oxidative stress, stabilizing mitochondrial NAD⁺ pools, enhancing autophagy, attenuating chronic low-grade inflammation, restoring neuromuscular junction integrity, and improving the quantity and functionality of muscle stem cells [103]. Lifelong overexpression of *Nampt* in aged mice preserves intramuscular NAD⁺ levels and sustains exercise capacity [104]. Osteoarthritis (OA) and osteoporosis (OP) are prevalent musculoskeletal disorders that share common risk factors, including aging, metabolic dysfunction, and systemic inflammation. Extensive preclinical and clinical evidence highlights NAMPT’s critical involvement in OA and OP progression. Reduced NAMPT expression and impaired osteogenic differentiation are observed in bone marrow-derived mesenchymal stem cells (BM-MSCs) from elderly humans and murine models. Genetic ablation of *Nampt* in BM-MSCs promotes adipogenic differentiation and exacerbates age-related bone loss. Conversely, NAMPT overexpression in aged mice ameliorates senescence-associated phenotypes in BM-MSCs and enhances osteogenic potential. Pharmacologically, the NAMPT activator P7C3 mitigates BM-MSC senescence, demonstrating therapeutic promise for counteracting age-related osteoporotic progression [105]. SIRT1 plays a pivotal role in bone metabolism and osteoporosis pathogenesis. Mechanistically, NAMPT promotes osteogenic differentiation in MC3T3-E1 cells via the SIRT1 signaling axis while amplifying lipopolysaccharide (LPS)-induced inflammatory responses. These findings position NAMPT as a potential therapeutic target for modulating inflammation-associated bone resorption [106, 107]. However, some studies reveal that NAMPT interacts with the innate immune receptor TLR4, driving inflammatory and catabolic cascades in cartilage and bone. Moreover, NAMPT correlates with multiple pathological hallmarks of OA and OP [108]. Collectively, the role of NAMPT in these diseases exhibits context-dependent duality, exerting both pro-homeostatic and detrimental effects. Consequently, future investigations must rigorously delineate NAMPT’s bidirectional regulatory mechanisms to evaluate its dual utility as a biomarker and therapeutic target.

Aging is accompanied by metabolic dysregulation in the body. Thermogenic beige adipocytes have emerged as potential therapeutic targets for mitigating metabolic diseases; however, their metabolic advantages diminish with aging. Studies have demonstrated that treating mice with estrogen (E2) promotes beige adipogenesis under cold exposure, enhances energy expenditure, and improves glucose tolerance. NAMPT plays a pivotal role in E2-induced beige adipocyte formation by suppressing age-related endoplasmic reticulum (ER) stress. Genetic or pharmacological potentiation of NAMPT signaling increases the population of perivascular adipose progenitor cells and restores beige adipogenesis, whereas NAMPT signaling deficiency inhibits this process. These findings elucidate mechanisms governing beige adipocyte regulation and underscore the critical involvement of the E2-NAMPT-controlled ER stress pathway in this context [109]. Further investigations have explored the metabolic consequences of adipose-specific NAMPT ablation in aging female mice. While NAMPT-deficient mice do not develop obesity during aging, they exhibit severe insulin resistance in skeletal muscle, cardiac tissue, and WAT, accompanied by hyperinsulinemia and hypoadiponectinemia. Mechanistically, NAMPT loss markedly reduces the expression of peroxisome proliferator-activated receptor gamma (PPAR-γ) target genes in WAT. Treatment with the PPAR-γ agonist rosiglitazone restores adipose mass and ameliorates metabolic derangements in NAMPT-deficient mice. These results highlight the essential role of NAMPT and its downstream NAD⁺ pool in preserving adipose tissue functionality and systemic metabolic homeostasis [110].

The decline of NAMPT and NAD⁺ is strongly implicated in cerebral aging and the pathogenesis of neurodegenerative disorders. Depletion of NAMPT in cortical projection neurons induces motor dysfunction and mortality in mice [111]. NAD⁺ exhaustion via *Nampt* knockout in hippocampal neurons drives neurodegeneration through mitochondrial impairment [112]. Furthermore, NAMPT deficiency triggers time-dependent loss of dopaminergic neurons and Parkinson’s disease-like phenotypes in murine models [113]. Conversely, NAMPT overexpression mitigates ischemia-induced neuronal death through dual inhibition of caspase-dependent and independent apoptotic signaling pathways, alongside suppression of mitochondrial damage and dysfunction [114]. Secreted eNAMPT in peripheral circulation is associated with aging-related diseases. Research demonstrates that SIRT1-mediated secretion of eNAMPT from adipose tissue regulates hypothalamic NAD⁺ levels and locomotor activity in mice. On the other hand, SIRT1-dependent deacetylation of intracellular NAMPT enhances its secretory propensity in adipocytes. A NAMPT mutant in mice causes SIRT1-mediated deacetylation at lysine 53 (K53), resulting in augmented eNAMPT enzymatic activity and secretion [40]. Additionally, extracellular vesicles (EVs) isolated from exercise regimens and enriched with eNAMPT can modulate NAD⁺ abundance and SIRT1 activity in recipient cells. Systemic delivery of eNAMPT via exercise-released EVs may represent a physiological mechanism to mitigate age-associated NAD⁺ decline [38].

Cellular senescence represents a state of irreversible cell cycle arrest. While transient senescence contributes to development, wound healing, and tumor suppression, chronic senescence drives inflammation, tissue dysfunction, and age-related pathologies. The senescence-associated secretory phenotype (SASP), a collection of bioactive molecules secreted by senescent cells, including cytokines, chemokines, and proteolytic enzymes, amplifies with aging, releasing pro-inflammatory cytokines and perpetuating inflammation and senescence [115]. Chronic exposure to SASP-derived inflammatory cytokines suppresses NAMPT expression while elevating CD38 levels, both of which synergistically induce systemic NAD⁺ depletion. This global NAD⁺ decline precipitates cellular/tissue dysfunction and age-related pathophysiology. However, the role of NAMPT-regulated NAD⁺ biosynthesis in SASP modulation remains to be fully elucidated. Emerging evidence indicates that NAMPT controls pro-inflammatory SASP independently of senescence-associated growth arrest. Mechanistically, NAMPT promotes pro-inflammatory SASP via NAD⁺-mediated suppression of AMPK kinase activity, which relieves p53-dependent inhibition of p38-MAPK, thereby enhancing NF-κB transcriptional activity [116]. Intriguingly, while NAMPT levels increase in senescent cells, intracellular NAD⁺ pools remain unaltered. Instead, senescent cells actively secrete NAMPT via exosomes as a SASP component. The eNAMPT exists as a catalytically active dimer that can be internalized by neighboring cells, elevating their NAD⁺ levels and extending cellular longevity. Thus, NAMPT exhibits context-dependent duality in SASP: functioning both as an NAD⁺-synthesizing enzyme and a SASP factor that modulates the progression of aging-associated diseases [117]. CD38, now recognized as the predominant NAD⁺-degrading enzyme (NADase) in mammalian tissues, increases with aging, driving NAD⁺ depletion and mitochondrial dysfunction. Studies reveal that CD38 impairs mitochondrial fitness by suppressing SIRT3 deacetylase activity, a key regulator of NAD⁺-dependent mitochondrial homeostasis [118]. Pharmacological inhibition of CD38 with the selective inhibitor 78c improves survival in progeroid mice, ameliorates aging-associated metabolic and structural deficits, and extends health span and lifespan in naturally aged male mice [119].

**Fig. 3:**
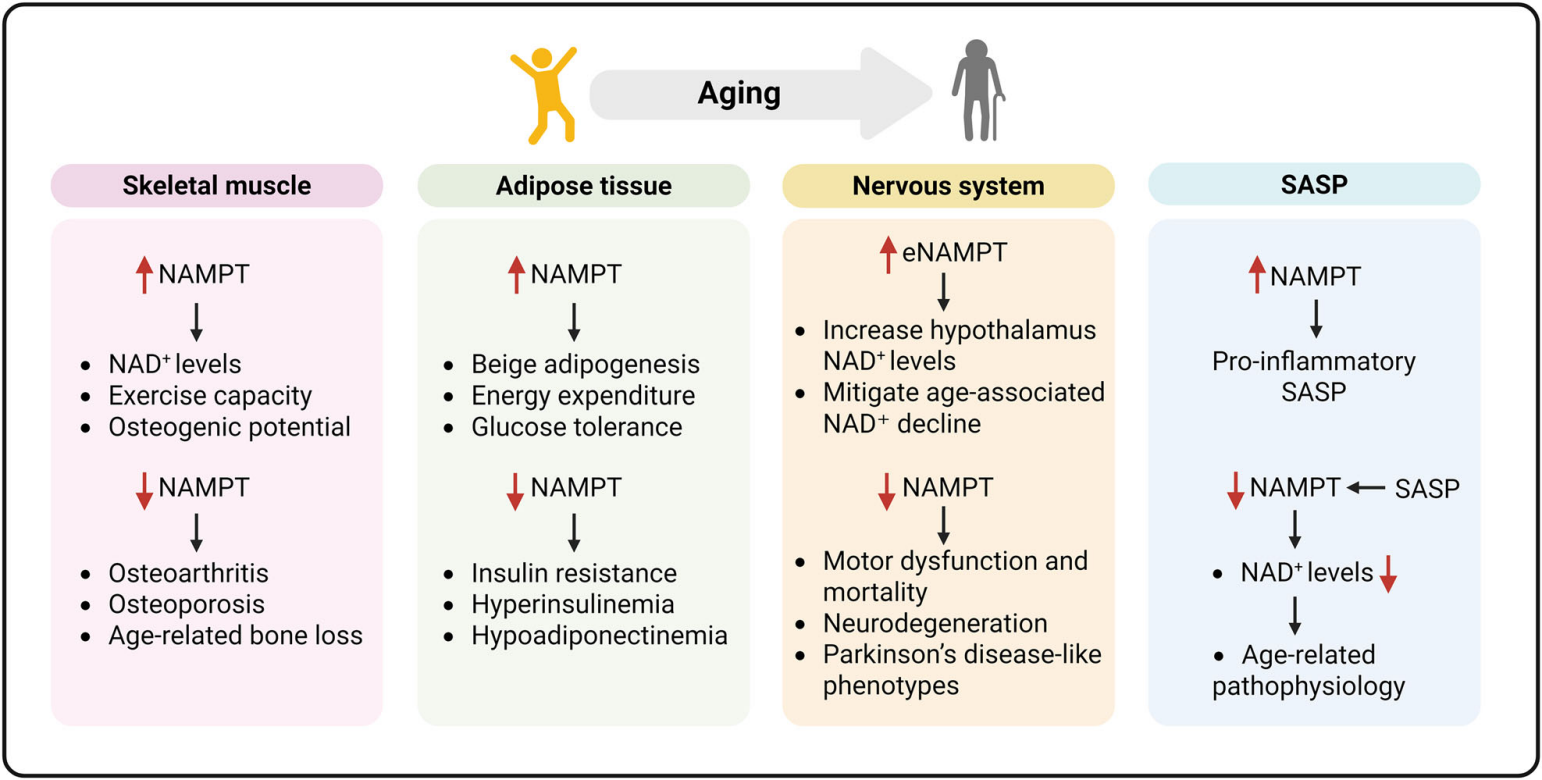
Tissue-specific impacts of NAMPT dysregulation on age-related disorders. Declining NAMPT levels are critically linked to the progression of age-related diseases across multiple tissues, including skeletal muscle, adipose tissue, and the nervous system. In skeletal muscle, reduced NAMPT activity contributes to osteoarthritis, osteoporosis, and age-related bone loss. Within adipose tissues, NAMPT is essential for beige adipogenesis, and its deficiency drives metabolic derangements such as insulin resistance, hyperinsulinemia, and hypoadiponectinemia. In the nervous system, NAMPT depletion induces motor dysfunction, neurodegeneration, and Parkinson’s disease-like phenotypes in murine models, ultimately leading to premature mortality. Restoring NAMPT levels in these tissues ameliorates corresponding aging-associated pathologies. Furthermore, NAMPT exhibits inhibitory effects on the senescence-associated secretory phenotype (SASP), underscoring its dual role in modulating both tissue-specific dysfunction and systemic inflammatory cascades during aging.

In summary, NAMPT plays a central role in regulating cellular metabolism and inflammatory responses through its modulation of NAD⁺ homeostasis, with its declining activity emerging as a key driver of aging. Targeting the NAMPT-NAD⁺ axis represents a promising therapeutic strategy to mitigate aging and treat age-related pathologies. Future research should prioritize molecular-level precision in modulating aging processes via this pathway, advancing senotherapeutic interventions toward clinical translation (Fig. 3).

### NAMPT and cancer

Recent advances have provided a wealth of information to better understand the pathogenic role of NAMPT in regulating the proliferation, migration, survival, and drug resistance of tumor cells, as well as its influence on the immune status of the tumor microenvironment [120–122]. NAMPT-driven deregulation of metabolism in cancer cells represents a vulnerability that can be therapeutically exploited to benefit tumor patients. Low-dose clinical NAMPT inhibitor OT-82 depletes NAD⁺ and inhibits cell growth through a metabolic defect characterized by significant impairment of glycolysis and diminished oxidative phosphorylation, leading to profound ATP depletion, irreversible necrotic cell death, and complete tumor regression when administered according to the clinical schedule in rhabdomyosarcoma [123]. Super enhancers specific to triple-negative breast cancer drive the transcriptional activation of cancer dependency genes through chromatin looping. Specifically, enhanced NAMPT expression promotes NAD⁺ and ATP metabolic reprogramming, which is critical for filopodia formation and tumor metastasis [124].

NAMPT-mediated NAD⁺ salvage dictates mitochondrial homeostasis and oxidative phosphorylation activity, supporting the optimal anti-tumor immunity of immune cells. However, in human hepatocellular carcinoma tissues, NAMPT expression and NAD⁺ levels were significantly downregulated in tumor-infiltrating NK cells, correlating negatively with patient survival. Mechanistically, lactate accumulation in the tumor microenvironment partially contributes to the transcriptional repression of NAMPT in NK cells. NAMPT deficiency impaired NK cell anti-tumor immunity and accelerated tumor growth [125]. In addition, tumor cell-secreted eNAMPT reprograms CD10^+^ALPL^+^ neutrophils through neurotrophic receptor tyrosine kinase 1, maintaining them in an immature state and inhibiting their maturation and activation. These neutrophils exhibit strong immunosuppressive activity by inducing apparent “irreversible” exhaustion of T cells, thereby contributing to tumor immune escape from durable anti-PD-1 treatment [126]. NAMPT is also highly expressed in a distinct subset of tumor-associated macrophages (TAMs) with immunosuppressive activity. A high NAMPT gene signature in SPP1^+^ TAMs is associated with poorer prognostic outcomes in colorectal cancer patients. In this context, NAMPT deficiency led to HIF-1α destabilization, resulting in reduced TAM polarization and a significant decrease in the efferocytosis activity of macrophages. This deficiency enhanced STING signaling and the induction of type I IFN-response genes, thereby contributing to anti-tumoral immunity by potentiating cytotoxic T cell activity in the tumor microenvironment [127]. NAMPT also regulates tumor immune evasion through a CD8^+^ T cell-dependent mechanism. NAMPT-mediated NAD⁺ biosynthesis maintains the activity and expression of methylcytosine dioxygenase TET1 through α-ketoglutarate. Concurrently, IFNγ-activated STAT1 promotes TET1 binding to IRF1, regulating IRF1 demethylation, which leads to downstream PD-L1 expression and contributes to immunosuppression in tumors. However, tumors with high NAMPT expression were more sensitive to anti-PD-L1 treatment, and NAD⁺ augmentation enhanced the efficacy of anti-PD-L1 antibodies in immunotherapy-resistant liver cancer [128]. On the other hand, neutralizing eNAMPT with the monoclonal antibody C269 activated CD8^+^IFNγ^+^GRZB^+^ T cells and reduced the immunosuppressive phenotype of regulatory T cells, thereby restoring antitumoral immune responses, decreasing tumor size, and reducing the number of lung metastases in triple-negative breast cancer [28]. RICTOR, as an essential subunit of mTORC2, plays a tumor-suppressing role during the early adaptation phase of BRAFV600E melanoma cells to targeted therapy. Reduced RICTOR expression resulted in elevated NAMPT expression and increased mitochondrial respiration, contributing to the intrinsic tolerance of drug-naïve tumor cells to BRAF/MEK inhibition and fostering a BRAF inhibitor-resistant phenotype. This identified NAMPT as a potential therapeutic target in tumors with low RICTOR expression [129].

Targeting NAMPT, which is selectively activated by oncogenic kinases to which malignant cells become “addicted,” may represent a novel therapeutic approach to cancer, either as an alternative or, more likely, as a complementary strategy to direct inhibition of the kinase enzymatic domain. This potential therapy, which simultaneously inhibits and metabolically “starves” oncogenic kinases, may not only lead to higher response rates but also delay, or even prevent, the development of drug resistance, which is frequently observed when kinase inhibitors are used as single agents. For example, NAMPT is selectively overexpressed in anaplastic T-cell lymphoma carrying the oncogenic kinase NPM1::ALK (ALK + ALCL). NPM1::ALK induces the expression of the NAMPT-encoding gene, with STAT3 acting as a transcriptional activator. NAMPT inhibition functionally impairs key metabolic and signaling pathways in ALK + ALCL cells, notably including the enzymatic activity and oncogenic function of NPM1::ALK itself. Consequently, NAMPT inhibition induces cell death in vitro and suppresses ALK + ALCL tumor growth in vivo [130]. Of note, most newly developed compounds are designed to inhibit the enzymatic activity of NAMPT, neglecting other crucial aspects like the extracellular role of NAMPT and the capability of alternative enzymes to counteract NAMPT dysfunction-mediated NAD⁺ depletion. It is essential to consider these aspects to develop innovative strategies and design inhibitors and molecules that show promise as anti-cancer agents [131–133]. In one instance, circulating nicotinic acid riboside (NAR), a non-canonical form of niacin absent in culture media, antagonizes the efficacy of NAMPT inhibitors by fueling NAMPT-independent, nicotinamide riboside kinase 1-dependent NAD⁺ synthesis in tumors. Depleting blood NAR through nutritional or genetic manipulations was synthetically lethal to tumors when combined with NAMPT inhibitors, suggesting a rationale for simultaneously targeting NAR metabolism and NAMPT as a therapeutic strategy in neuroendocrine carcinoma [134]. Additionally, the use of an NAPRT inhibitor as an adjuvant improved the efficacy of NAMPT inhibitors and reduced their required dose and associated toxicity. Coadministration of these inhibitors synergistically inhibited tumor growth by targeting cancer stem cells [135].

**Fig. 4:**
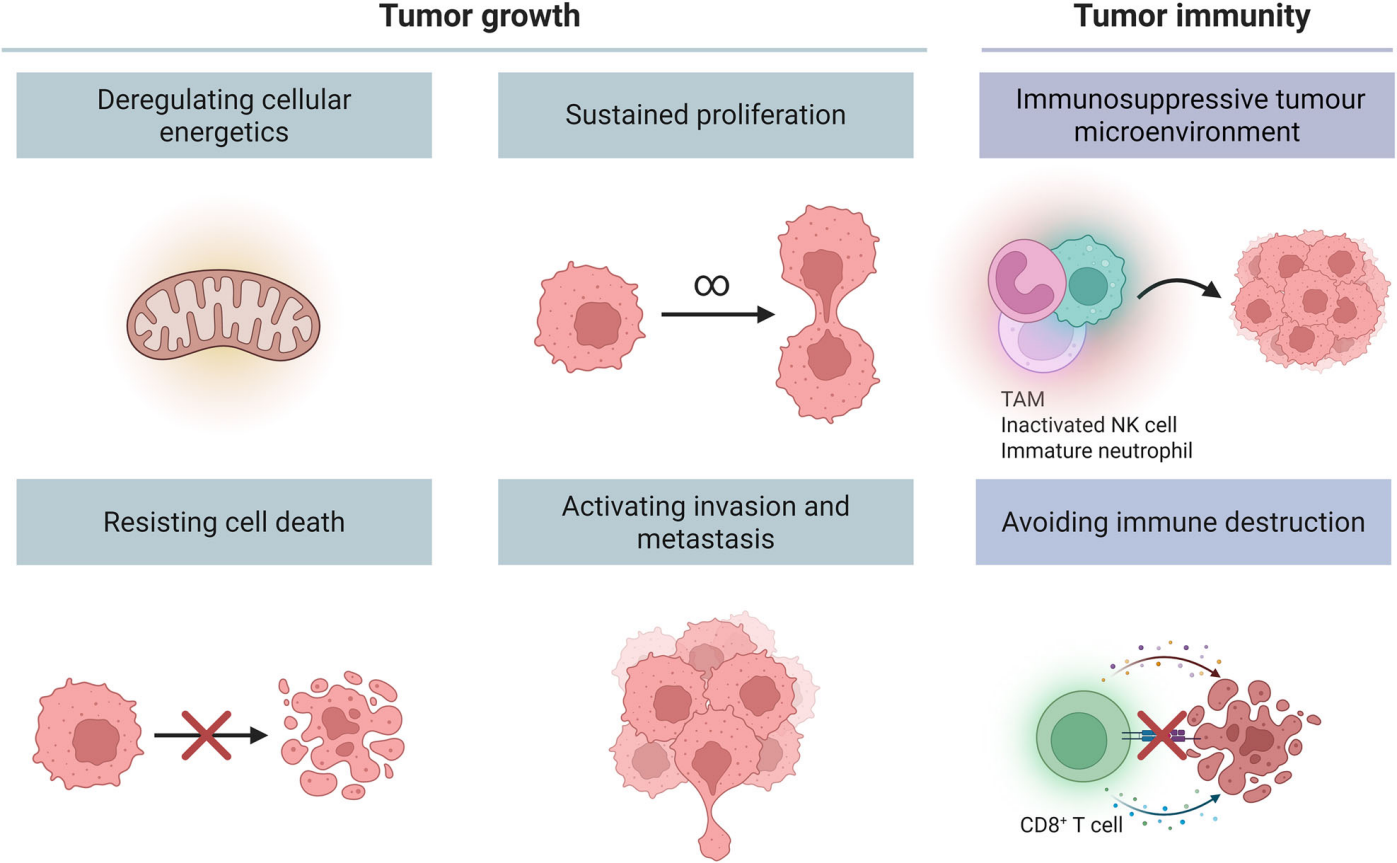
NAMPT and NAD^+^ metabolism in tumor growth and immunity. In tumor growth, NAMPT-mediated NAD⁺ synthesis contributes to cellular energetics, enabling sustained proliferation, resistance to cell death, and activation of invasion and metastasis. In the context of tumor immunity, NAMPT influences the immune microenvironment by promoting an immunosuppressive tumor milieu, characterized by the activation of tumor-associated macrophages (TAMs), inactivation of natural killer (NK) cells, and the presence of immature neutrophils. These immune alterations enable the tumor to evade immune destruction, including the suppression of CD8⁺ T cell-mediated cytotoxicity.

Taken together, NAMPT plays a critical role in cancer by regulating tumor cell proliferation, migration, survival, and drug resistance, as well as influencing the immune status of the tumor microenvironment. By modulating NAD⁺ metabolism, NAMPT represents a promising therapeutic target, and its inhibition can effectively disrupt tumor cell metabolism and induce tumor regression. NAMPT also plays a significant role in tumor immune evasion, impacting immune cell functions and regulating anti-tumor immune responses. Therapeutic strategies targeting NAMPT, particularly in combination with other treatments, have the potential to overcome resistance to current therapies and enhance treatment efficacy (Fig. 4).

## Conclusions and perspectives

NAD⁺ metabolism is a cornerstone of cellular energy balance and physiological regulation. As a coenzyme in redox reactions, NAD⁺ supports fundamental processes such as glycolysis, OXPHOS, and fatty acid oxidation. Beyond its role in energy metabolism, NAD⁺ participates in numerous non-redox functions, including post-translational modifications mediated by sirtuins, PARPs, and other NAD⁺-dependent enzymes. The intricate network of NAD⁺ biosynthesis, salvage, and degradation pathways ensures the maintenance of cellular NAD⁺ pools, with NAMPT playing a crucial role in the regeneration of NAD⁺ from NAM. However, dysregulation of NAD⁺ metabolism is linked to several pathological conditions, including metabolic disorders, aging, and cancer. In particular, NAD⁺ levels decline with age and in response to cellular stress, leading to impaired mitochondrial function, DNA damage, and compromised cellular homeostasis. Given its essential role in maintaining cellular integrity and function, NAD⁺ has become a target for therapeutic intervention. Strategies to boost NAD⁺ levels, such as the supplementation of NAD⁺ precursors (e.g., NR and NMN), have shown promise in preclinical models and some clinical trials (Table 1). The salvage synthesis pathways of NAD⁺ present promising targets for therapies aimed at increasing NAD⁺ levels in vivo. Specifically, NAMPT activators have been proposed as potential therapeutic agents to elevate tissue NAD⁺ concentrations. For instance, the neuroprotective compound P7C3 has been shown to enhance NAMPT activity and raise NAD⁺ levels in doxorubicin-treated human cells, indicating its potential as a therapeutic for aging and age-related diseases, including neurodegeneration [136]. Furthermore, enhancing NAMPT activity in MSCs with P7C3 has been shown to improve their therapeutic efficacy in treating inflammatory conditions [22]. More recently, SBI-797812, a small molecule NAMPT activator effective in the nanomolar range, was identified as a potent agent. This compound not only boosts NAMPT-mediated NMN production in vitro but also increases NAD⁺ levels in vivo [137]. NAMPT inhibitors have also been proposed as potential therapeutic agents to inhibit cancer progression. For example, treatment with KPT-9274, a NAMPT inhibitor, reduced the conversion of saturated fatty acids to monounsaturated fatty acids, a process catalyzed by the stearoyl-CoA desaturase enzyme, leading to apoptosis in acute myeloid leukemia cells [138]. Additionally, targeting NAMPT with the clinically relevant inhibitor FK866, in combination with platinum-based chemotherapy, presents a promising therapeutic approach by suppressing therapy-induced senescence-associated cancer stem-like cells [139].

**Table 1:** Evidence of benefits of supplemental NAD^+^ precursors in humans.

Physiological or pathological state	Improved by	Health benefits/effects	Trial number
Middle-aged healthy adults	NMN [140–142] NAM [143]	Increase walking distance and aerobic capacity during exercise training, delay the biological age of blood, and show potential in alleviating arterial stiffness. Additionally, improve insulin sensitivity and glucose tolerance, reduce fatigue, and enhance mental focus and motivation	NCT04823260 UMIN000045205 ChiCTR2000035138 NCT04483011
Older adults	NMN [144, 145] NR [146]	Prevent the loss of physical performance, improve fatigue and sleep quality, augment neuronal NAD^+^ levels, and modify biomarkers related to neurodegenerative pathology	UMIN000038097 UMIN000047871 NCT02921659
Overweight or obesity	NMN [147] NR [148]	Increase circulating NAD^+^ levels, reduce total LDL and non-HDL cholesterol, body weight, and diastolic blood pressure, and affect skeletal muscle acetylcarnitine metabolism and sleeping metabolic rate	NCT02835664
Non-alcoholic fatty liver disease	NR [149]	Decrease circulating levels of the liver enzymes alanine aminotransferase, gamma-glutamyltransferase, and the toxic lipid ceramide 14:0	NCT03513523
Prediabetes	NMN [150]	Increase muscle insulin sensitivity, insulin signaling, and remodeling	NCT03151239
Parkinson’s disease	NR [151, 152]	Improve MDS-UPDRS clinical scores, induce transcriptional upregulation of processes related to mitochondrial, lysosomal, and proteasomal functions in blood cells and skeletal muscle, and decrease the levels of inflammatory cytokines in serum and cerebrospinal fluid	NCT03816020 NCT05344404
Peripheral artery disease	NR [153]	Enhance walking performance	NCT03743636
Chronic obstructive pulmonary disease	NR [154]	Reduce the production of sputum interleukin-8	NCT04990869
Ataxia telangiectasia disease	NR [155]	Enhance motor coordination and eye movements	NCT04870866
Chronic kidney disease	NR [156]	Improve systemic mitochondrial metabolism and lipid profiles	NCT03579693
Heart failure	NR [157]	Enhance mitochondrial respiration and attenuate the pro-inflammatory activation of peripheral blood mononuclear cells	NCT03727646
Glaucoma	NAM [158]	Enhance visual field mean deviation and inner retinal function, and reduce deterioration	ACTRN12617000809336

*NAD*^+^ nicotinamide adenine dinucleotide, *NMN* nicotinamide mononucleotide, *NAM* nicotinamide, *NR* nicotinamide riboside, *LDL* low density lipoprotein, *HDL* high-density lipoprotein, *MDS-UPDRS* movement disorder society unified Parkinson’s disease rating scale.

Looking forward, a deeper understanding of the molecular mechanisms underlying NAD⁺ metabolism is essential to fully harness its therapeutic potential. Future research should focus on several key areas, including tissue-specific NAD⁺ dynamics, as NAD⁺ metabolism is highly compartmentalized with distinct cellular pools in the cytoplasm, mitochondria, and nucleus. Investigating the tissue- and organ-specific roles of NAD⁺ and its intermediates will provide insights into how to target NAD⁺ metabolism more precisely for therapeutic purposes. Additionally, NAMPT is a central enzyme in the NAD⁺ salvage pathway, and understanding its regulation in response to cellular stress, aging, and disease will be crucial for developing strategies to modulate NAD⁺ levels during disease. While short-term studies have shown that NAD⁺ precursors can boost NAD⁺ levels and improve cellular function, the long-term safety and potential risks of chronic NAD⁺ supplementation remain underexplored. Investigating these aspects is essential to assess the broader therapeutic applications of NAD⁺ modulation. Finally, recent studies have highlighted the involvement of NAD⁺ metabolism in immune responses and inflammation, opening new avenues for therapeutic approaches for diseases involving immune dysregulation, such as autoimmune diseases and cancer. In summary, NAD⁺ and its central enzyme NAMPT are crucial to maintaining cellular health, and their dysregulation contributes to a wide range of diseases. With the growing interest in NAD⁺ as a therapeutic target, future studies will be pivotal in unlocking the full potential of NAD⁺ metabolism in medicine, ultimately leading to the development of effective therapies for age-related diseases and beyond.
